# Guideline for incorporating the Delphi method in the evaluation of nursing theories

**DOI:** 10.1590/1518-8345.4157.3387

**Published:** 2021-05-21

**Authors:** Manuela Campos Gomes Borel, Rafael Oliveira Pitta Lopes, Maira Buss Thofehrn, Maria Miriam Lima Da Nbrega, Cristina Arreguy-Sena, Marcos Antnio Gomes Brando

**Affiliations:** 1 Universidade Federal de Juiz de Fora, Faculdade de Enfermagem, Juiz de Fora, MG, Brazil.; 2 Universidade Federal do Rio de Janeiro, Campus Maca, Maca, RJ, Brazil.; 3 Universidade Federal da Paraba, Centro de Cincias da Sade, Joo Pessoa, PB, Brazil.; 4 Universidade Federal do Rio de Janeiro, Escola de Enfermagem Anna Nery, Rio de Janeiro, RJ, Brazil.

**Keywords:** Knowledge, Models, Theoretical, Nursing Theory, Validation Study, Decision Making, Nursing, Conhecimento, Modelos Tericos, Teoria de Enfermagem, Estudo de Validao, Tomada de Deciso, Enfermagem, Conocimiento, Modelos Tericos, Teora de Enfermera, Estudio de Validacon, Toma de Decisiones, Enfermera

## Abstract

**Objective:**

to describe a guideline for the use of the Delphi method to evaluate nursing theories, from the perspective of internal validation.

**Method:**

a methodological study, targeted at the development of a guideline for the use of the Delphi method in the evaluation of nursing theories.

**Results:**

the Delphi method, principles of collective wisdom and levels of proficiency are used in the production of a guideline for organizing, searching, selecting and coordinating the activities of theoretical evaluators in teams. It distinguishes three phases for the theoretical evaluation process: Preparatory Phase (PP); Intermediate Phase (IP) and Theory Evaluation (TE) phase, incorporating Delphi-type selection procedures; search, selection and classification of judges/evaluators for the theory; definition of criteria for carrying out rounds and maintenance or removal of units of the theory evaluated.

**Conclusion:**

the developed guideline was able to adapt the elements of the Delphi method as a favorable strategy for the internal validation of nursing theories.

## Abstract (pt)

**Objetivo:**

descrever uma diretriz de uso do mtodo Delphi para avaliao de teorias de enfermagem, na perspectiva de validao interna.

**Mtodo:**

estudo metodolgico, dirigido ao desenvolvimento de uma diretriz para o uso do mtodo Delphi na avaliao de teorias de enfermagem.

**Resultados:**

o mtodo Delphi, os princpios da sabedoria coletiva e os nveis de proficincia so utilizados na produo de uma diretriz para organizao, busca, seleo e coordenao de atividades de avaliadores tericos em equipes. Distingue trs fases para o processo de avaliao terica: fase preparatria (FP); fase intermediria (FI) e fase de avaliao da teoria (AT), incorporando procedimentos de seleo do tipo de Delphi; busca, seleo e classificao de juzes/avaliadores da teoria; definio de critrios para a realizao de rodadas e de manuteno ou descarte de unidades da teoria avaliada.

**Concluso:**

a diretriz elaborada foi capaz de adequar os elementos do mtodo Delphi como uma estratgia favorvel validao interna de teorias de enfermagem.

## Abstract (es)

**Objetivo:**

describir una directriz para el uso del mtodo Delphi en la evaluacin de teoras de enfermera, desde la perspectiva de la validacin interna.

**Mtodo:**

estudio metodolgico, dirigido a la elaboracin de una directriz para el uso del mtodo Delphi en la evaluacin de teoras de enfermera.

**Resultados:**

el mtodo Delphi, los principios de sabidura colectiva y los niveles de competencia se utilizan en la elaboracin de una directriz para organizacin, bsqueda, seleccin y coordinacin de las actividades de los evaluadores tericos en equipos. Distingue tres fases para el proceso de evaluacin terica: fase preparatoria (FP); fase intermedia (FI) y fase de evaluacin terica (ET), con la incorporacin de procedimientos de seleccin tipo Delphi; bsqueda, seleccin y clasificacin de jueces/evaluadores de la teora; definicin de criterios para la realizacin de rondas y mantenimiento o disposicin de unidades de la teora evaluada.

**Conclusin:**

la directriz desarrollada logr adaptar los elementos del mtodo Delphi como estrategia favorable para la validacin interna de las teoras de enfermera.

## Introduction

The theoretical construction through the theory-research strategy is a process initiated in the elaboration of an explicit theory in the phases of conceptual development and operationalization, later on, needing to proceed to the confirmation or non-confirmation phases, until reaching practical application conditions^(^ 1 ^)^. In the applied disciplines, empirical validation is emphasized to produce a judgment about usefulness and application.

The total undertaking of theoretical construction requires judgments to be carried out to estimate the value of a good theory, which is that rich in theoretical virtues, as science philosophers point out. Among these virtues, we can highlight the observance of singularity, falsification, parsimony, prediction, explanation, conservationism, capacity for generalization, fecundity, internal consistency, empirical wealth and abstraction^(^ 2 ^)^.

In identifying the good theory, an internal perspective can guide validation, the judgment of the intrinsic elements or by the external perspective, the judgment using the empirical test^(^ 3 ^)^. In nursing, internal validation is commonly referred to as theory evaluation which aims to determine the appropriateness of its use and the epistemological approach^(^ 4 ^)^. Depending on the formal criteria to be used, the evaluation can incorporate analysis or theoretical breakdown.

Despite the relevance of theory evaluation and the existence of dozens of structured and systematic criteria for its realization, it is still unusual to verify the application of these criteria in international literature. External validations are more common in empirical studies with statistical analyses or literature reviews^(^ 4 ^)^.

There is a continuous interest in the production of middle-range theories to describe better, explain, predict or prescribe the phenomena, facts, events or interventions with which nursing deals in daily life. However, these theories, together with those of a specific situation, are the most rarely evaluated^(^ 4 ^)^.

If, on the one hand, this continuous movement contributes to the progress of the discipline, on the other hand, it requires the availability of instruments and guidelines that promote good theoretical development practices. Therefore, access to resources that can assist in the program of elaboration, validation, refinement and theoretical application is indispensable.

The internal validation (evaluation) of a theory requires, at the same time knowledge of the theory and a high level of meta-theoretical knowledge. For such reason, it is difficult to find experienced and available meta-theorists to perform this task. Whenever possible, the coordination of this task is difficult or, even, is a complicated procedure the identification of an analyst considered proficient by the application of epistemologically consistent criteria.

Given the difficulty in locating meta-theoretical experts, the principles and criteria of collective wisdom or crowd wisdom can be useful for the construction of guidelines, methods or techniques that guide the formation of a team, capable of developing the task of theoretical evaluation with the same or superior result, compared to that of a single meta-theoretical expert. In the crowd wisdom theory, criteria such as independence, decentralization, diversity and aggregation would guide the constitution of groups, in which the aggregate decision would surpass that of the specialist, separately^(^ 5 ^)^.

In this way, analysts, not necessarily experts in meta-theory, act as judges for the content, the structure and other criteria to be judged. From the aggregate judgment, consistent results are achieved that allow for the theory evaluation to be carried out successfully. However, guidelines, methods or techniques with this conformation are not available for use with nursing theories.

Presumably, the Delphi method is adequate to evaluate a nursing theory supported by the crowd wisdom criteria, demonstrating which groups can judge adequately under conditions of uncertainty, defining the fundamental concepts, judging and adding the collective value of the ideas^(^ 5 ^-^ 7 ^)^. It has been used to deal with issues not clarified by experimental approaches in which the opinion of a group has value to clarify them, therefore, being compatible with internal validation^(^ 6 ^)^.

However, its application for this purpose is scarce. Its use was identified in the literature only in a theory of the education-informatics interface in the evaluation of the criteria of importance, precision and clarity, parsimony or simplicity, understanding, operationalization, empirical validity, fruiting and application^(^ 8 ^)^. The methodological description in the study mentioned above does not provide enough elements for its use in the evaluation of nursing theories with formal criteria, usually applied in the discipline^(^ 4 ^)^.

In Brazil, the Delphi method has helped in addressing practical problems such as trend indication, obtaining consensus on a program or intervention, expert opinion for comparing treatments and, more widely, in the construction of tools for evaluation and in the creation and validation of instruments^(^ 9 ^)^. The adaptation of the method for nursing theories evaluation remains a potential that has not yet been explored, even given its innovative character. This article was prepared given the scarcity of research studies and the potential from the development of a guideline.

The article aims to describe a guideline for the use of the Delphi method to evaluate nursing theories, from the perspective of internal validation.

## Method

This research is a methodological study for developing a guideline for the use of the Delphi method in nursing theories evaluation, indicating procedures for organizing, searching, selecting and coordinating the activities of theoretical evaluators in teams. The criteria of collective wisdom and levels of proficiency^(^ 5 ^)^ were the reference basis and its elaboration took place in Rio de Janeiro, RJ, Brazil, between the months of November and December 2019.

The elements used in the methodological frameworks for the design, construction and testing of guidelines were incorporated, highlighting the following: selecting the topic and scope; adapting a prototype of a theoretical evaluation strategy guideline, using the Delphi method; group formation for development; systematic search for evidence; analysis and synthesis of available evidence and elaboration of the recommendation^(^ 10 ^)^.

The specific procedures for developing the guideline were the following: a simple review of manuscripts on the use of the Delphi method in theories evaluation and other applications; interpreting nursing theory evaluation methods^(^ 4 ^,^ 11 ^-^ 12 ^)^; selection of the complementary material on the topic of collective wisdom; compiling and interpreting the results of using a prototype of a theoretical evaluation guideline developed in a masters thesis by one of the authors, incorporating features of the Delphi method; elaborating the guideline, taking into account the principles of construction of guidelines in health and the necessary adaptations to the theoretical-philosophical object; discussion and review by the authors; final elaboration of the guideline with diagramming interpretation of nursing theory evaluation methods.

The prototype developed in the masters thesis had the following stages: (a) selection of the *experts*; (b) contact with experts and invitation for participation by those selected; (c) electronically sending the instrument to those who agreed to participate; (d) appreciation of theory evaluation items based on an agreement Likert scale; (e) receiving the answers; (f) qualitative and quantitative analysis of the results; (g) adaptation of the content for a new round of theoretical evaluation; (h) forwarding with *feedback* containing the data that led to the modification or maintenance of the items to perform a new evaluation; (i) receiving the answers to the adapted instrument; (j) analysis of the data from the second version; (l) final construction by consensus; (m) grammatical and orthographic review and (n) closing the theoretical evaluation.

The masters dissertation that incorporated the use of the prototype evaluated the Theory of Professional Links^(^ 13 ^)^ by Meleis theoretical evaluation strategy^(^ 14 ^)^. The study that applied the prototype of the guideline respected the ethical principles of research contained in Resolution 466/2012 of the National Health Council, obtaining an approval opinion from the Research Ethics Committee, under number 3.237.583.

## Results

**Figure 1:**
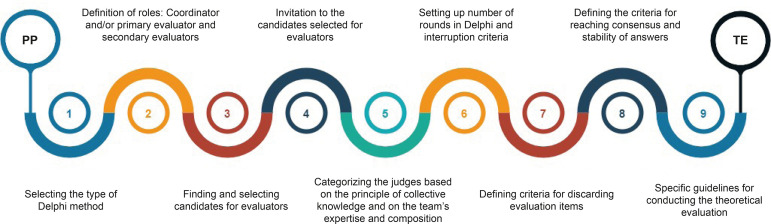
Outline of the guideline for incorporating the Delphi method in nursing theories evaluation. Rio de Janeiro, RJ, Brazil, 2019

Encompassing the Delphi method in the nursing theories evaluation, the guideline has three phases: Preparatory Phase (PP), Intermediate Phase (IP) and Theory Evaluation (TE) phase. This study details the intermediate phase, as shown in Figure 1.

In the preparatory phase (PP) the theory to be evaluated is chosen and the strategy to be employed is selected from the alternatives available in the literature.

In the intermediate phase, nine procedures related to the use of the Delphi method are outlined. The first procedure is related to the type of Delphi to be used, influenced by the level of the theory to be evaluated and by its application maturity.

In the second procedure, the coordination role of the theoretical evaluation is defined, which can be accumulated with the condition of a primary evaluator. This can be done by a member of the theory development team or by another researcher with consistent knowledge about it, being responsible for preparing and conducting the evaluation process. The role of the primary evaluator is to provide help to the secondary evaluators in accessing the analysis materials, with the ability to produce a preliminary report with results of the task performed that serves as a primary document intended for consultation by the other evaluators. Otherwise, the secondary operate as judges from the primary document or by performing a primary evaluative function.

The third procedure is the location and selection of candidates for evaluators/judges. The search can be performed in platforms that contain electronic resumes, the following being used as filters: nationality, academic background, language and professional performance, among others necessary. Appraisal of publications, projects or research studies is recommended, as well as verification of the performance time in the area for the careful selection of the candidate for evaluator. The selection of candidates for secondary evaluators is usually difficult, in the absence of classification criteria for a presumed meta-theoretical expertise.

So, based on collective wisdom^(^ 5 ^)^, the teams of evaluators (judges) must have diversity in their expertise levels. The expertise of the judges is analyzed by criteria in five domains, namely: Educational training in nursing theories; Professional experience in the theoretical area; Meta-theoretical experience and knowledge; Dissemination of knowledge produced on the topic of nursing meta-theories or theories and Peer-recognition of expertise on the topic of nursing meta-theories or theories (Figure 2).

**Figure 2:** Criteria for classifying the presumed level of expertise of evaluators of nursing theories. Rio de Janeiro, RJ, Brazil, 2019

Domains and criteria for categorizing theory evaluators/judges	Score
1. Educational training in nursing theories:	
a) PhD in Nursing or related areas and theoretical or meta-theoretical thesis	4 points
b) Master's degree in Nursing or related areas and theoretical or meta-theoretical thesis	3 points
c) PhD in Nursing with a thesis on other topics	2 points
2. Professional experience in the area of the theory to be evaluated:	
(a) More than four years of experience in teaching, research or clinical practice in the area/theme of the theory to be evaluated and, at least, one year in teaching theories or theoretical or meta-theoretical nursing research	4 points
b) More than four years of experience in teaching, research or clinical practice in the area/theme of the theory to be evaluated	3 points
c) Between two and four years of experience in teaching, research or clinical practice in the area/theme of the theory to be evaluated or, at least, one year in teaching theories or theoretical or meta-theoretical research in nursing	2 points
3. Meta-theoretical experience and knowledge:	
a) Elaboration or orientation of more than one nursing theory or meta-theory	4 points
b) Elaboration or orientation of a nursing theory or meta-theory	3 points
c) Elaboration or orientation of, at least, one non-nursing theory or meta-theory	2 points
4. Dissemination of knowledge produced on the topic of nursing meta-theories or theories:	
a) Authorship in more than one article published in an international standard indexed journal with a high impact factor (for Brazil: Qualis A1) on the theme of nursing theory or meta-theory	4 points
b) Authorship of an article published in an international standard indexed journal with a high impact factor (for Brazil: Qualis A2 to A4) on nursing theory or meta-theory	3 points
c) Authorship of, at least, one article published in a national indexed journal with a medium impact factor (for Brazil: Qualis B1) on the theme of nursing theory or meta-theory	2 points
5. Peer-recognition of expertise on the topic of nursing meta-theories or theories:	
a) More than one participation as a guest (lecturer, speaker, commentator, an instructor/professor in a course or short course) in a scientific event to teach a theme related to nursing theories or meta-theories	4 points
b) Participation as a guest (lecturer, speaker, commentator, an instructor/professor in a course or short course) in an event to teach a theme related to nursing theories or meta-theories	3 points
c) Participation as a listener/participant/student in a completed event or course on nursing theories or meta-theories	2 points

The candidate for evaluator/judge has their level of expertise ranked by the score obtained by the sum of the items of the five domains. The maximum score for each domain is 4 points and the minimum is 2. When the evaluators do not meet any of the criteria for a domain, they receive a zero score. The total score ranges from 0 to 20, 4 points being the minimum arbitrated value to consider the candidate suitable to be a member of the theoretical evaluation team.

The level of presumed expertise for a candidate for evaluator is established by the score obtained from the analysis of the five domains. Five levels are proposed, namely: beginner; advanced beginner; competent; proficient and expert.

The classification of a candidates presumed expertise level is established from their total score obtained (see Figure 1).

The definition of the minimum number of evaluators in the team will depend on the sum of the individual points of each evaluator and on the mixed composition that guarantees members with at least two different levels of expertise (Figure 3).

**Figure 3:** Classification of the level of presumed expertise for the evaluators according to the total score obtained and definition of the number of evaluators in the team. Rio de Janeiro, RJ, Brazil, 2019

Assumed level of expertise of the evaluator	Score required to be fit to the level	Criteria for defining the team, according to the expertise points
Beginner	Minimum sum of 4 points	(a) Team of two evaluators: The sum of the evaluators' points must be at least 35 points (b) Team of three evaluators: The sum of the evaluators' points must be at least 36 points (c) Team of four evaluators: The sum of the evaluators' points must be at least 48 points (d) Team of five evaluators: The sum of the evaluators' points must be at least 60 points (e) Team of six or more evaluators: Apply the following equation:
Advanced beginner	Sum between 5 and 10 points	
Competent evaluator	Sum between 11 and 14 points	
Proficient	Sum of scores between 15 and 17 points	
Expert	Sum of scores greater than or equal to 18 points	

Source: Brando, MA, 2019

For example, by applying the proposed equation, composing a team of seven evaluators by calculating the total required points of the sum of the evaluators will require approximately 74 points. Exemplifying, an appropriate configuration would include six advanced beginner evaluators with 10 points each and a competent evaluator with 14 points. Obviously, other configurations that respect the minimum score for the team can be applied. In addition to the total score, the requirement for mixed teams in terms of expertise levels aims to guarantee the criterion on diversity of judges.

Knowing that the losses in the face of invitations and during initial Delphi rounds are common, it is recommended to select a higher number of judges required for the minimum composition of the teams, seeking to maintain proportionality between the levels.

The fourth procedure is the invitation to the selected candidates, based on written or electronic communication and respecting ethical research principles.

The fifth procedure involves the reapplication of the criteria for categorizing the evaluators/judges by their expertise levels and adjustments to the composition of the teams considering refusals to participate.

The sixth procedure is the planning of rounds and interruption criteria. This planning considers the level of abstraction, the number of concepts and the complexity of the theory to be evaluated. The number of judges developing the evaluations is also noteworthy, as is the consideration regarding the number of criteria to be evaluated in the theory. The interruption of the rounds must be supported by the explicit judgment on the part of the evaluators after reaching an evaluative consensus or constitution of a multiplicity of ideas in the dissent. Another decision is to establish or not, *a priori*, a maximum number of rounds. This decision refers more to time available for the task than to the evaluative judgment.

Subsequently, to guide the evaluators, explicit criteria must be established for discarding items in each round. The items of a theory subjected to evaluation are its components like concepts, assumptions, suppositions, statements, and model schemes. Therefore, the evaluators must be certain that the decision to exclude is driven by the selected strategy and not, only, by their freely-expressed personal opinions. Their function is to judge a given theory item against the evaluation criteria established in the strategy.

The eighth procedure encompasses the definition of the consensus scope and of the stability of the answers. The consensus can be verified by formal measures of agreement, measures of central tendency, percentage of agreement, and measure of central tendency within a specific interval, among others^(^ 15 ^)^. The use of a five-point Likert scale can be planned with two purposes: (1) to verify the agreement of secondary evaluators with the result of the primary evaluation or (2) to organize the secondary evaluations in assertions that will be submitted to agreement analysis in a later round.

Even when scales are applied, it is recommended to guarantee free editing fields so that the evaluators/judges can express their suggestions, recommendations and detailed appraisals.

The ninth procedure is to provide specific guidance on the theoretical evaluation strategy. When the coordinator or primary evaluator deems it necessary, complementary and specific training on the content of the strategy can be carried out.

## Discussion

Theoretical evaluation is able to provide elements about a good theory, with several formal and systematic criteria available in the literature^(^ 2 ^,^ 11 ^)^. However, human resources with the competence and knowledge required to properly develop the process of judging theoretical virtues are not always available. And, in this respect, by using the principle of collective wisdom by consensus or dissent, the Delphi method can multiply the groups expertise, further expanding the universe of alternatives for the evaluation^(^ 15 ^-^ 16 ^)^. Likewise, it assists in the coordination of the process.

Through the evaluation, relationships and links of concepts are perceived, allowing the reviewer to verify the theorys strengths and limitations; identifying the need for new elements of the theory or improving the existing ones and, as a final goal, determining the potential contribution of the evaluated theory for the scientific knowledge^(^ 11 ^)^.

Unlike the theory analysis that decomposes a theory to examine its parts or components^(^ 4 ^)^, theoretical evaluation also judges them. However, even a theory judged to be good can prove to be inadequate in its descriptive, explanatory, predictive or prescriptive value from its confirmation or application. This places internal validation as a relevant stage, although not terminal of a theoretical development program.

Theories that violate the virtues of a good theory are more difficult to refute and tend not to, actually, contribute to knowledge^(^ 17 ^)^. The inadequacy of elements and constructions hinders theoretical evaluation and testing. Thus, it is fundamentally important to plan internal and/or external validation as part of a more comprehensive program. Using the guideline herein presented may avoid the expenditure of resources, when collaborating in the identification of theories that do not have sufficient virtues to support validation by field research.

The reasons for the reduced use of nursing theory evaluation strategies through formal systematic criteria are uncertain^(^ 4 ^)^. However, influence can be attributed to the difficulty in obtaining evaluators with sufficient epistemic authority to judge the meta-theoretical items of internal validation. It is supposed that the strategies linked to collective wisdom can overcome this problem of dependence on the expert with substantial advantages^(^ 18 ^)^.

The Delphi method is based on the John Deweys assumptions, emphasizing anonymous communication between individuals with expertise in a given topic, with the goal of seeking the opinion of experts in an iterative and structured way and usually seeking to achieve a consensual position^(^ 15 ^,^ 19 ^)^. The freedom and observance of the judges personal opinions guarantees the independence criterion of collective wisdom^(^ 5 ^)^.

Regarding the use in research studies, although it is used predominantly in mixed and quantitative, it has its qualitative application and even in the construction of practical theories, in the context of community organization^(^ 15 ^)^. Theory evaluation is a qualitative process permeated by subjectivity and by standards, conducts and codes of the evaluator^(^ 8 ^)^.

The Delphi method can coordinate these qualitative characteristics of the evaluation process, dealing with personal variables of the independence criterion, making the most of group work. It can be used for interpretation, for predictions and for obtaining recommendations of the evaluation developed^(^ 8 ^)^.

In choosing the Delphi method, the most common approach is the traditional one, also being referred to as normative or of consensus. It aims to reduce variance in the estimates and biases among experts. However the Delphi Policy or Policy of dissent, seeks to obtain a wide range of opinions, but without seeking consensus^(^ 16 ^)^.

For the theoretical evaluation, consensus Delphi is the most likely indication; however, the use of dissent can be recommended for theories of high originality, conceptual density, complexity and theoretical abstraction or when it is difficult to determine the consensus criteria. Additionally, one of the goals of the evaluation can be to explore the contradictions in the production of definitions or theoretical proposals.

Regarding the characteristics of the theory, consensus Delphi can be indicated for those of micro- or middle-range with conceptualization described in more than one empirical study or to evaluate partially disseminated, tested or used theories.

Supposedly, for consensus Delphi the composition of teams with a high number of evaluators is only justified when it is difficult to obtain evaluators with higher levels of expertise, because it is challenging to obtain consensus in groups of many components. On the other hand, it is assumed that the dissent approach benefits from the composition of larger teams and with a wide range of proficiency levels, tending to broaden the debate from different perspectives and to bring original elements that differ from the original theory and from the primary evaluation.

Panels with more participants tend to have lower answer rates, with an estimated reduction of 0.08 percentage points for each added participant^(^ 20 ^)^. A number of 5 to 20 experts are indicated if it is a recommendation based exclusively on the characteristics of the Delphi method^(^ 20 ^)^. Studies on the development and application of Core Outcome Set (COS) have used the Delphi method to determine which results to measure, with the predominance of Delphi panels of up to 50 people^(^ 20 ^)^.

In the theoretical evaluation it is challenging to establish a minimum and maximum number of evaluators/judges, due to its philosophical character and abstract epistemological nature inherent to theorization. For example, for new or poorly disseminated theories, it can be difficult to have many secondary evaluators with adequate expertise. On the other hand, large teams of beginner evaluators may not have knowledge of a meta-theoretical nature, causing a dispersion of perspectives that would hinder the aggregation of ideas. In this case, the guideline seeks to circumvent the limits by combining a balance between the criterion of diversity of the principle of collective wisdom and the expertise required for theoretical evaluation^(^ 4 ^-^ 5 ^)^.

The prototype of the guideline included four evaluators with three different expertise levels, and three secondary evaluators who together collectively summed 36 points (14, 13, and 9 individual points). According to the expertise points, the criteria for defining the team were useful for the composition of this small group, as the configuration of fewer participants guaranteed the maximum answer rate, as expected for this panel size^(^ 20 ^)^. The differences in training levels and stories of the evaluators ensured the decentralization criterion^(^ 5 ^)^. However, whenever possible, it is recommended to assemble teams with five or more judges.

Patricia Benners model^(^ 21 ^-^ 22 ^)^ with its five levels of competence acquisition was the basis for creating the judges criteria of expertise in the guideline and sought to recognize the professional experience as an essential component for validation. The wide dissemination of studies by these authors and their criteria helped in the definition. There are more sophisticated models of aggregation rules to define the composition of the team, for example, the Contribution Weighted Model (CWM) that weighs the prognosis based on the relative performance of each judge and the accuracy of the group^(^ 18 ^,^ 23 ^)^.

The contributions of evaluators/judges have knowledge, experiences, and particular points of view in the evaluation of the theory. The iterative process of the Delphi method can allow that, in the rounds, the obscure criteria of the evaluation can be clarified or modified, through a careful interpretation of the answers of the secondary evaluators, by the coordinator. The composition of teams with different levels of competence guarantees the diversity criterion of collective wisdom^(^ 5 ^)^.

The studies commonly apply two to three rounds for the Delphi method^(^ 19 ^)^. However, the multiple criteria to be evaluated, the high number and diversity of profiles of the evaluators may require more rounds to reach consensus. It is desirable to plan a minimum according to the number of evaluators, to ensure that an excessive effort to manage the task results does not fall on the Delphi coordinator, compromising their quality.

The scope level of theories can influence the definition of criteria to be evaluated by judges; for example, when a given middle-range theory is evaluated as a model, even more specific and empirical criteria can be used^(^ 12 ^)^. However, this does not, directly, interfere with the nature of the Delphi method as a strategy.

The decision to reach consensus among judges is a type of mechanism to meet the criteria of aggregating collective wisdom, transforming individual judgments into a teams decision^(^ 5 ^)^. The consensual decision can start from the evaluators own opinion that a consensus was reached; however, it is recommended that this does not happen automatically after completion of the Delphi technique^(^ 19 ^)^.

It is necessary to specify which conditions are required for reaching consensus when the decision is qualitative. When quantitative measurement procedures are adopted, establishing the measures and cut-off points will be used to establish the degree of agreement or disagreement, compatible with the consensus or dissent^(^ 19 ^)^.

There are no mandatory rules for consensus building, but the five-point Likert scale is the most common among the scales used to estimate disagreements or agreements^(^ 24 ^)^. It makes it possible to check the degree of agreement for each item or set. For consensus reach estimates using the Likert scale, formal agreement measures such as the Kappa statistic can be applied, to verify the judges concordant judgment on the elements of the theory.

Usually, the percentages of agreement adopt the value of 0.8 or 80% as a minimum cut-off point^(^ 15 ^)^. However, the researcher can consider other cut-off points, supported by evidence or by a consistent recommendation. An explicit statement on the reach of consensus is indicated with an indication of the reasons that were considered in decision-making.

Another useful measure that can be used on the data obtained by the Likert scale is the content validity coefficient. The Aiken coefficient and its caudal probability table can be used to indicate the validity of a particular item evaluated by several judges, estimating a consensus. It can also be applied to judge the validity, by a single judge, of the content for all the items of the theory. The coefficient range varies from 0 to 1, with higher values indicating validation^(^ 25 ^)^.

Despite the literature generally recommending the use of quantitative scales to signal consensus, qualitative justifications must be added, especially when the recommendation is for the items invalidity^(^ 24 ^,^ 26 ^)^. The simple exclusion of an item can make the whole theory incoherent or illogical. This is because units of a theory play roles and have different relevance in the theoretical structure.

For example, the exclusion of an assumption can de-characterize the theory as a whole, since this typology of element functions as premises not given to the empirical test. Thus, its removal negates the ideas that guided the theorists themselves in constructing the theory. On the other hand, proposal type units are submitted, precisely, to generate testing hypotheses in empirical validation studies; therefore, they are naturally subjected to exclusion or maintenance after evidence obtained from experimentation or field research^(^ 1 ^)^.

It is highlighted that, from the evaluation of the Theory of Professional Links^(^ 27 ^-^ 28 ^)^, emerging factors demanded changes to criteria not detailed when the prototype was elaborated, which contributed to the deepening for the creation of the guideline presented in this article.

The study is not limitation-free. The focus of any research using the Delphi method will always be obtaining high-quality answers from a selection of expert individuals^(^ 29 ^)^. However, the internal validation of a theory deals with theoretical-philosophical criteria that can make it difficult, for a secondary evaluator, to produce or judge the quality of the answers by the nature of the object evaluated and by the judgment property to be performed. For example, the conceptual definition is one of the elements of a theory, evaluated in its semantics, logic, and context^(^ 14 ^)^. Notably, it can be difficult to make a good answer judgment for such a complex construct, given such properties.

The limitation for the subjectivity of the judges judgment in theories evaluation must be confronted with the philosophical root of the theorist and of the evaluator. Critical-social, hermeneutic or new pragmatism roots tend to deal with greater fluidity in the face of different perspectives, including exploring them in consensus or in dissent. On the other hand, as it requires greater objectivity of reality, post-positivism requires more stable, generalizable or measurement criteria^(^ 30 ^)^. In this last philosophical root, methods such as structural equation modeling, factorial analysis and multiple regressions may be the best choice for theory evaluation, obviously with criteria closer to external validation^(^ 4 ^)^.

Among the contributions for the advancement of scientific knowledge, the study adds an unexplored dimension of the incorporation of evaluators of different levels of meta-thematic expertise in the task of theoretical evaluation, including incorporating guidelines for the phases of this process. Given the growth in developing middle- and micro-range nursing theories and of a specific situation, with the consequent training of new theorists, the guideline can facilitate the validation process for the new theories, making up a solid base of disciplinary knowledge^(^ 4 ^,^ 31 ^)^.

On the other hand, higher levels of meta-theoretical expertise tend to require long years of training in this field, being more common to be verified in academia and among senior researchers. By exploring the principle of diversity of expertise levels for theoretical evaluation, the study encourages the creation of teams of different expertise levels, promoting cooperation and the circulation of knowledge to those involved in this process.

Finally, the application of the Delphi method in nursing theory evaluation must be clearly understood as different from the search for consensus on events, phenomena, facts, technologies, conducts or any other fundamentally empirical elements. In the empirical Delphi method, the removal of an item can have minimal implication; however, in the theory, the judgement of the inadequacy of central suppositions or concepts can place the whole theory in the condition of inadequate. Obviously, the main goal of the evaluation is to identify a good theory, which implies judging the adequacy of its components; however, this procedure must be performed with extreme caution by the evaluators, understanding that, in a theory, there is hierarchy and relationship between the elements.

## Conclusion

The guideline developed was able to adapt the elements of the Delphi method as a favorable resource for the internal validation of nursing theories, enhancing it with the incorporation of judges with different views of the world, experiences, scientific knowledge, and creativity. The criteria displayed in the guideline adapt and articulate the proficiency levels of the evaluators with the principle of crowd wisdom, serving as a guide for the selection and composition of teams of judges, as well as facilitating the coordination of the theoretical evaluation work. Due to its innovative character, the guideline can instrumentalize nursing meta-theorists and, possibly, speed up the process of applying theories in practice.

The use of a guideline prototype in the evaluation of middle-range nursing theory, the Theory of Professional Links, brought satisfactory results that presume its feasibility and pointed out ways for refinement.

It is understood that it is essential that other researchers replicate its use in the evaluation of grand- and micro-range theories for future adjustments and updates of the guideline, also adopting evaluation strategies by formal criteria different from the one used in the prototype.
